# Redesign and Validation of Sisom, an Interactive Assessment and Communication Tool for Children With Cancer

**DOI:** 10.2196/mhealth.5715

**Published:** 2016-06-24

**Authors:** Susann Arvidsson, Britt-Mari Gilljam, Jens Nygren, Cornelia Maria Ruland, Trude Nordby-Bøe, Petra Svedberg

**Affiliations:** ^1^ School of Health and WelfareR.N. Halmstad University Halmstad Sweden; ^2^ Region Halland Halmstad Sweden; ^3^ The Centre for Shared Decision Making and Collaborative Care Research (CSDM) Oslo University Hospital Oslo Norway; ^4^ University of Oslo Oslo Norway

**Keywords:** cancer, children, communication, mobile app, participation, validation

## Abstract

**Background:**

Children with cancer undergo intensive and long treatment periods that expose them and their families to a number of difficult physical, mental, and social challenges. Empowering children by actively involving them in their care can help them to cope with these challenges. It can, however, be difficult for children to be involved and talk about their illness experiences in a “traditional” conversation with health care professionals, especially for younger children. Sisom (Norwegian acronym “Si det som det er” or “Tell it how it is”) is an interactive computer-based assessment and communication tool to give children (aged 6-12 years) with cancer a “voice” in their care. Because of technological advances and widespread use of mobile devices Sisom had to be redesigned to better meet the needs of children of today.

**Objective:**

To redesign Sisom for use on mobile devices and to validate and adapt it for use in a Swedish population of children with cancer.

**Methods:**

A user-experience design was used. Content adaptation included forward-backward translation by Swedish and Norwegian translators. Healthy children (n=5), children with experiences of cancer treatment (n=5) and their parents (n=5), and pediatric nurses (n=2) were then involved in culturally adapting Sisom to the Swedish context. The iterative low- and high-fidelity evaluation was supported by a think aloud method, semistructured interviews, and drawings to capture children’s views of Sisom. The redesign and evaluation continued until no further changes or improvements were identified by the participants or the researchers.

**Results:**

Children, parents, and pediatric nurses offered many suggestions for improvements to the original version in terms of content, aesthetics, and usability of Sisom. The most significant change that emerged through user input was a modification that entailed not using problem-focused statements in the assessment items. The parents and pediatric nurses considered the revised assessment items to be general and less diagnosis specific. The evaluation of aesthetics resulted in brighter colors and more positive and exciting details in the animations. The evaluation of usability included improvements of the verbal instructions on how to navigate in Sisom 2, and also that the answers to assessment items in Sisom 2 should be saved to provide the children with the option to pause and to continue answering the remaining assessment items at a later stage.

**Conclusions:**

Overall, this paper describes the process of using user-experience design with children in order to redesign and validate an interactive assessment and communication tool and how the outcomes of this process resulted in a new version, Sisom 2. All participants confirmed the usability and qualities of using the final version. Future research should be directed toward the implementation of Sisom 2 in clinical practice and to evaluate outcomes from individual and organizational levels.

## Introduction

Children who have life-threatening diseases such as cancer [1] undergo intensive and long treatment periods that expose them and their families to physical, mental, and social challenges. One way for children to cope with the challenges is to be involved in their own care [2]. Children also want to be listened to and wish to be included in discussions about their own care, in information sharing, and to be involved in minor decisions [2-5]. Many health care professionals recognize the need to include children in their own care [2,6,7]. However, children’s rights for participating is still unsatisfactorily applied in health care [8] and children’s own needs and preferences are often neglected in health care situations [5,9]. It is thus important to increase health care professionals’ awareness of the benefits of including children in their own health care [6,10]. New mobile devices have begun to transform the way health care professionals deliver health care and have the potential to increase children’s participation in their own care by providing appealing and easy-to-use digital services that can contribute to change communication patterns between children and health care professionals [11].

Children’s involvement in their own health care, regardless of disease, is a fundamental right [12]. When health care professionals acknowledge and respect children as actors and promote their opportunities to participate in health care settings together with their family, a child-centered approach is attained. It is important that such an approach contains both the adults’ perspective concerning the child’s best interest and the child’s own perspective concerning respect for what is important to her or him. When health care professionals and parents have a child’s perspective, they are attentive, sensitive, and supportive to the child, irrespective of the child’s age, gender, and so on [13]. Children have to be treated as individuals, and health care professionals and other adults must take into account that a child’s competence and preferences depend on situational circumstances [14]. It can, however, be difficult for children, especially younger ones, to talk about their experiences in a “traditional” conversation with health care professionals and with other adults. In order to overcome this problem, Sisom (Norwegian acronym “Si det som det er” or “Tell it how it is”) was developed. Sisom is an interactive computer-based assessment and communication tool for children with cancer [15,16] and heart disease [17] aged 6-12 years that gives children a “voice” in their care. With its child-friendly interface, Sisom helps children report their symptoms and problems, enabling health care professionals to better understand the children’s concerns and respond with appropriate care. The main theme in Sisom is a discovery journey among islands that the child can visit together with a personalized avatar. Sisom uses spoken text, sounds, animations, and intuitively meaningful metaphors and pictures to represent symptoms and problems, allowing even younger children who cannot yet read to understand and communicate [16].

Sisom is available in Norwegian, US and UK English, Spanish, Greek, and French. The previous versions of Sisom have been successfully tested in Norway and the United States, showing significant improvements in patient care and patient-provider communication: twice as many symptoms and problems were addressed when Sisom was used in pediatric consultations, without increasing consultation time [17]. Children also received significantly more information from the physicians, were asked more follow-up questions by the pediatric nurses, the parents and health care professionals communicated more often directly with the children, and the children participated more with information and in illness-related discussions more often [17]. The original version of Sisom has been widely tested for its usability [16-20]. However, because of technological advances and widespread use of mobile devices Sisom had to be redesigned to better meet the needs of children of today and their demands on aesthetics and usability of mobile apps available on mobile phones and tablets. The aim of this study was thus to redesign Sisom for use on mobile devices and to validate and adapt it for use in a Swedish population of children with cancer.

## Methods

### Design

The user-experience design [21] for redesigning and validating a revised version of Sisom included (1) forward-backward translation, (2) evaluation of the understanding of Sisom's symptom statements, and (3) iterative low- and high-fidelity evaluation [22], in a Swedish context with healthy children, children with experiences of cancer treatment and their parents, and pediatric nurses during a 10-month period in 2014-2015.

### Participants

For the forward-backward translation and the evaluation of the understanding of Sisom's symptom statements, purposive sampling was used to recruit Swedish translators (n=4), Norwegian translators (n=2), pediatric nurses working with the care of children with cancer (n=2), and healthy children (n=2). For the low- and high-fidelity evaluation, purposive sampling was used to recruit children with experiences of cancer treatment (n=5) and their parents (n=5), the same pediatric nurses (n=2) as previously involved, and healthy children (n=5) with some experience in medical treatment (eg, surgery, vaccination, treatment of eczema). The healthy children, who were recruited from academic researchers’ families, were used as proxies for children with experiences of cancer treatment, for evaluation of general understanding of content and usability that was not expected to be dependent on a prior experience of cancer treatment. The user-experience design could pose an impossible burden on children with experiences of cancer treatment. Therefore, the use of proxies was important in order to conserve access to a limited selection of children with a unique experience of cancer treatment to evaluate very general aspects of content, aesthetics, and usability. The pediatric nurses were recruited from a pediatric clinic in southern Sweden and were important participants because of their experiences and knowledge of caring for children with cancer with different symptoms or problems and also to judge if these symptoms or problems described in Sisom were relevant in a Swedish context. The children with experiences of cancer treatment and their parents were recruited by the pediatric nurses based on their judgment on whether the child felt well enough to ask for participation in the study. All children were selected based on sex, age (6-12 years), and literacy skills in Swedish. The child and family characteristics are presented in Table 1.

### The Original Version of Sisom

The original version of Sisom was an interactive computer-based communication tool with spoken texts, sounds, animations, and intuitively meaningful metaphors and pictures to represent symptoms and problems. Together with a self-selected avatar, the child sets out on a virtual journey from island to island (in total 5 islands: “At the hospital,” “About managing things,” “My body,” “Thoughts and feelings,” and “Things one can be afraid of”). Symptoms and problems (n=82) were placed on different islands: for example, physical symptoms were placed on the “My body” island, psychological problems on the “Thoughts and feelings” island, and so on.

Every symptom and problem was represented by an animation, a brief statement (also spoken by a cartoon nurse), and the assessment item “How much of a problem?” [16]. The child responded by selecting the level of severity on a 5-point Likert scale with cartoon faces (differently colored smileys) complemented in writing with “Not at all,” “A little,” “Some,” “A lot,” and “Don’t know.” In addition, the child could specify areas of pain, bruises, and rash on a body map [19]. The symptoms and problems used in Sisom were identified in a literature review [23] and the development of Sisom was carried out with clinicians (ie, physicians, nurses, psychologists), parents of children with cancer, healthy children, and children with cancer. Details of this development are described elsewhere [15,16].

### Data Collection and Procedure

The redesign of Sisom into Sisom 2 and adaptation to a Swedish context began with translation, where a Swedish version of Sisom was constructed through a forward-backward translation procedure [24,25]. Four persons with university degree, native Swedish speakers fluent in both Norwegian and Swedish, translated from Norwegian to Swedish. Then 2 persons with university degree, native Norwegian speakers fluent in both Norwegian and Swedish, and at that time with a limited knowledge of the original version of Sisom, retranslated to Norwegian. The backward translation, from Swedish to Norwegian, was used to improve the quality of the final Swedish version [24,25]. In the next step, healthy children and pediatric nurses contributed with an evaluation of the understanding of Sisom's symptom statements, in order to make the final translation culturally representative and child-friendly. This procedure was performed to also assess the level of comprehensibility and conceptual equivalence of the translation, to evaluate translation alternatives, to elicit any symptom statements that were difficult to understand at a conceptual level, and to identify symptom statements that could cause confusion [24,25]. A few minor differences emerged in the backward translation in comparison with the original version of Sisom and the evaluation of the understanding of Sisom symptom statements. These differences were more related to changes to a more child-friendly wording than a change of content. All differences were discussed in the research group and modified to achieve comprehensibility and conceptual equivalence [26].

**Figure 1:**
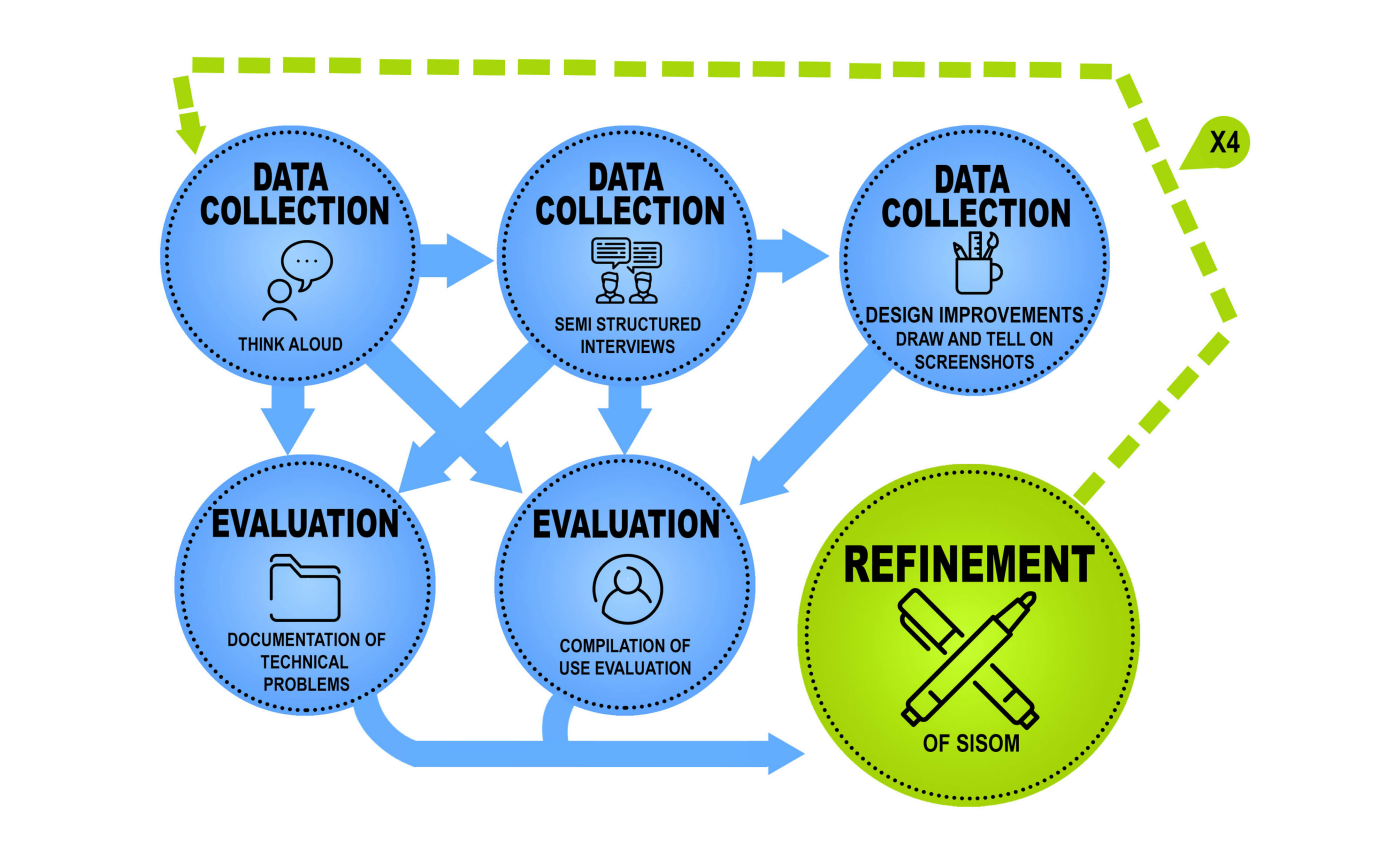
Outline of the iterative low- and high-fidelity evaluation process for redesign of Sisom, which resulted in 4 cycles in total.

In the next stage, iterative low- and high-fidelity evaluations were performed, consisting of 6 steps: (1) observation and evaluation where children were instructed to think aloud [27] when using Sisom; (2) semistructured interviews with each child and the parents separately, about what they liked and disliked about the design and aesthetics, the symptom statements, and assessment items posed; (3) paper screenshots of Sisom on which children were asked to draw or write suggestions for improvement of the graphics and pictures in Sisom; (4) documentation of technical problems; (5) compilation of use evaluation; and (6) data-driven refinement of the service toward Sisom 2. In steps 1-3 healthy children, children with cancer and their parents, and pediatric nurses participated, which resulted in a total number of 55 data collection encounters (see Table 2). These 3 steps were all audiotaped. Findings were discussed in the research team between the meetings with the children, and the latter’s ideas and opinions from steps 1-3 were used to guide revisions made by a graphical designer and a system developer in the team (steps 4-6). They drew the children’s ideas in the next rough versions of Sisom that were given back to the children for evaluation and further elaboration in the next meeting. This low- and high-fidelity evaluation (steps 1-6) was iterated until no further changes or improvements were identified by the participants or the researchers, resulting in 4 cycles in total (Figure 1).

**Table 1:** Child and family characteristics.

Characteristics			Healthy children	Children with cancer
Age in years, mean (SD)			8.4 (1.82)	8.2 (1.64)
Family household size, mean (SD)			4.8 (0.45)	5 (1.00)
**Characteristics of the children, n (%)^a^**				
Sex				
	Male		3 (60)	3 (60)
	Female		2 (40)	2 (40)
Cancer diagnosis				
	Acute lymphoblastic leukemia			2 (40)
	Brain tumor			1 (20)
	Ewing sarcoma			1 (20)
	Wilms tumor			1 (20)
Time since diagnosis, years				
	<2			1 (20)
	2-3			1 (20)
	4-5			3 (60)
Time since completed all cancer treatment, years				
	<1			1 (20)
	1-2			4 (80)
Parents view of child’s health status				
	Excellent			3 (60)
	Very good			2 (40)
	Good			0 (0)
	Fair			0 (0)
	Poor			0 (0)
**Characteristics of the families, n (%)^b^**				
Parental marital status				
	Married/cohabitation		6 (100)	10 (100)
	Divorced/separated		0 (0)	0 (0)
	Widowed		0 (0)	0 (0)
Parental education				
	< High school		0 (0)	1 (10)
	High school		1 (17)	7 (70)
	University		5 (83)	2 (20)

^a^ Healthy children, n=5; children with cancer, n=5.

^b^ Parents of healthy children, n=6; parents of children with cancer, n=10.

**Table 2:** Participants and the number of participants in each of the 4 cycles in the iterative low- and high-fidelity evaluation of Sisom.

Participants	Cycles, n					Total number of data collection encounters
	1	2	3	4		
3 boys (7-10 years) who have been treated for cancer	3	3	3	3		12	
2 girls (6-9 years) who have been treated for cancer	0	2	1	1		4	
3 healthy boys (6-11 years)	3	3	3	2		11	
2 healthy girls (8-9 years)	2	2	2	1		7	
5 parents of children who have been treated for cancer	3	5	4	4		16	
2 pediatric nurses	2	2	0	1		5	
Total sum	13	17	13	12		55	

### Data Analysis

The data (interviews, observations, and drawings) from the iterative low- and high-fidelity evaluation (steps 1-3) were evaluated by a qualitative content analysis [28]. This entails that each interview was listened to several times for familiarization and for gaining an overall impression of the data. Words or statements that related to the same central meaning were referred to as a meaning unit. The relationships between the meaning units, the observations, and the drawings were clustered and connected together. The analysis process resulted in 3 categories on a manifest level: evaluation of content, evaluation of aesthetics, and evaluation of usability. Representative quotations and drawings from the children are used in the results to illustrate the data in the categories. The analysis was carried out by the first author and evaluated by means of discussions between all the authors during the analysis process. Participant characteristics were analyzed with descriptive statistics using SPSS version 20.

### Ethical Considerations

All study procedures were performed with ethical permission from the regional ethical board in Lund (dnr: 2011-307). The children’s participation in the study was discussed with both parents and the child. The children, parents, and pediatric nurses were informed verbally and in writing about the study and that their participation was voluntary. They were assured confidentiality and that they could withdraw their consent to participate at any time without having to justify the reason and without it having any effect on their care.

## Results

The results from the redesign and validation process included 3 categories: evaluation of content, evaluation of aesthetics, and evaluation of usability that influenced the new version of Sisom.

### Evaluation of Content

**Figure 2:**
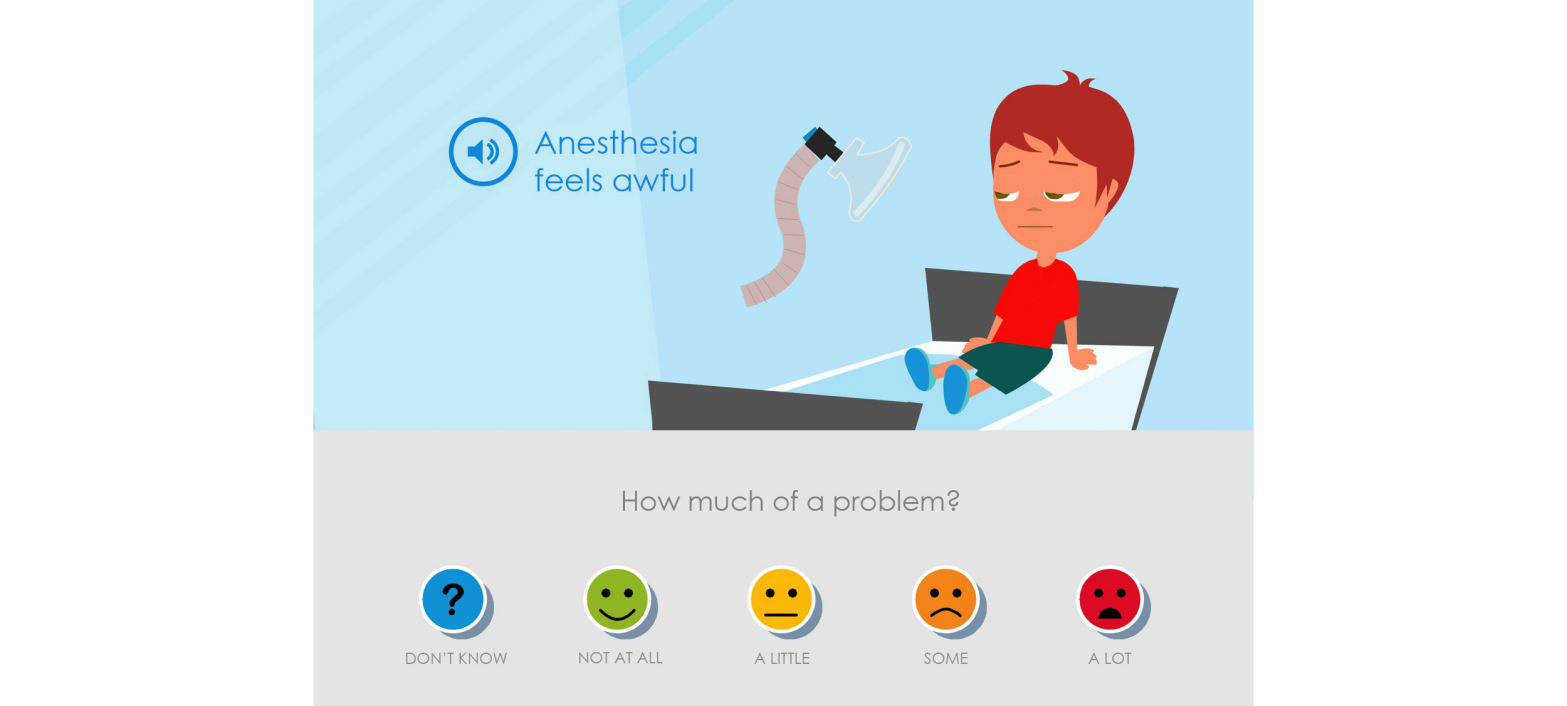
The parents and the pediatric nurses suggested that the statements should be more neutral than in this picture of the first version of Sisom 2.

When the children, parents, and pediatric nurses at the first low-fidelity evaluation of content reviewed and discussed the Swedish texts, most symptom statements were found to be appropriately adjusted to linguistic and cultural context, but some symptom statements needed further clarification. The parents and pediatric nurses also questioned why the symptom statements had such a negative or risk-accented phrasing, such as “Getting a tube feels awful” or “Sleeping problems.” They wanted, instead, that the symptom statements be more neutral and that children could use the cartoon smiley faces on the Likert scale to determine whether it was unpleasant or not and to rate the degree of severity (Figure 2).

**Figure 3:**
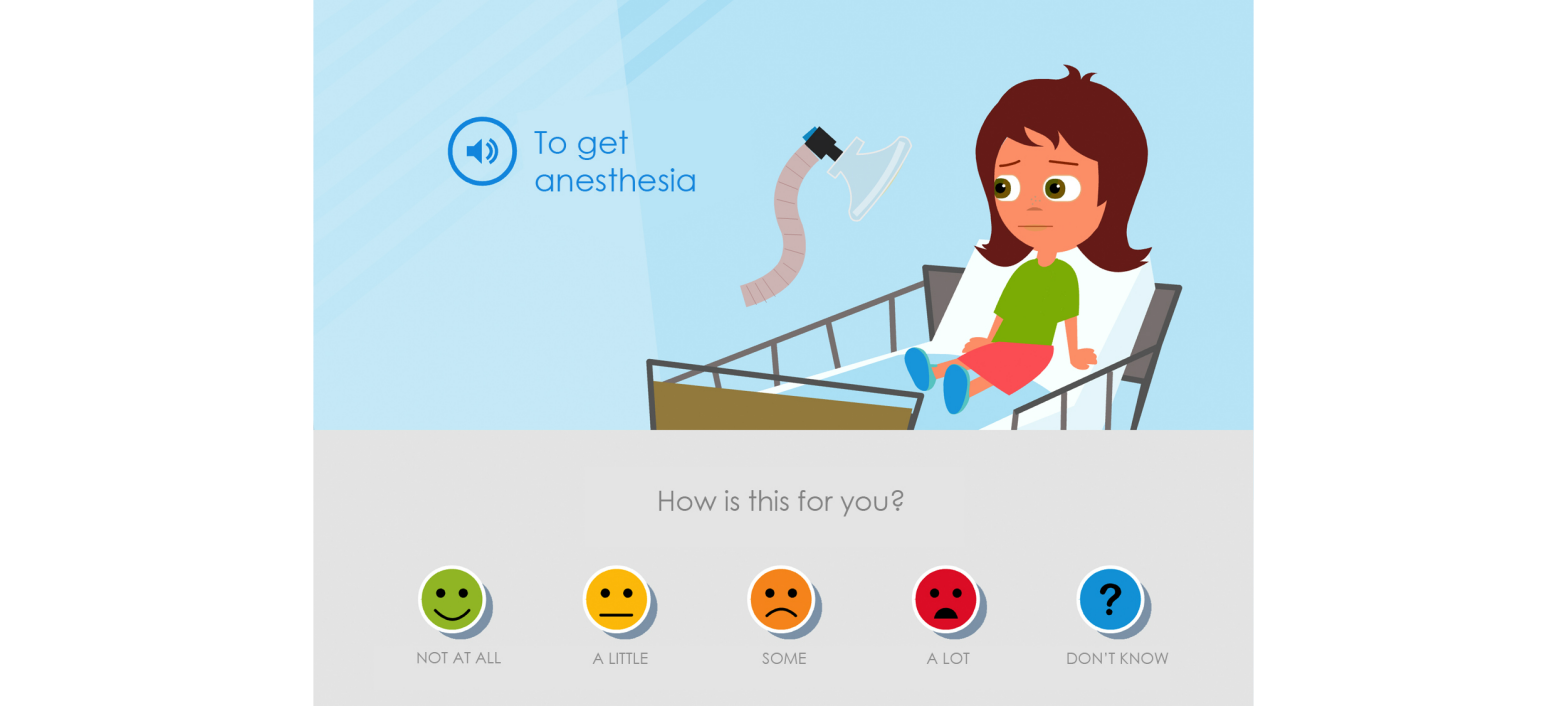
The parents and the pediatric nurses suggested that the statements should be more salutogenic and the statement was therefore adjusted from “Anesthesia feels awful” to just “To get anesthesia” and the assessment item was adjusted from “How much of a problem?” to “How is this for you?”.

The parents expressed that a modification of the symptom statements toward a more salutogenic perspective was important because when their child was ill they tried to focus on what their child could do and what generated joy in their lives. It had been important for them to not emphasize or focus on all the problems and difficulties that the child had. The symptom statements were thus modified to a more salutogenic and positive orientation instead of the perceived problem-focused orientation. This modification also entailed that the assessment item posed related to the Likert scale was adjusted from “How much of a problem?” to “How is this for you?”. Children could then choose from “No problem,” “A little,” “Some,” “A lot,” and “Don’t know” (Figure 3); that is, from “Anesthesia feels awful” and “How much of a problem?” to just “To get anesthesia” and “How is this for you?”. The other symptom statements and assessment items in Sisom 2 were rephrased according to this.

**Figure 4:**
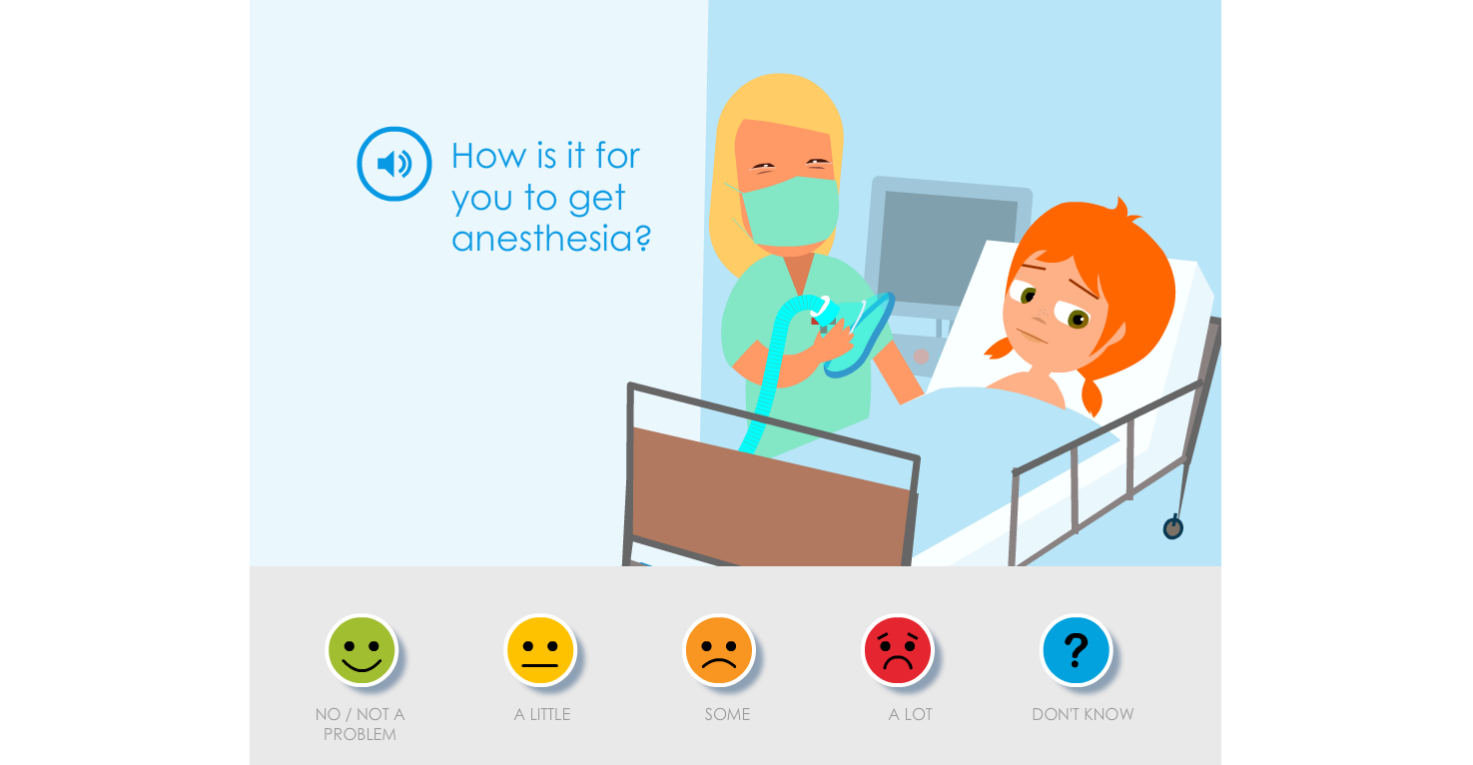
The picture shows the final outcome based on the children’s and parents’ suggestions that there should only be a single assessment item in a picture.

In the second evaluation, it became apparent that the children did not read the assessment item “How is this for you?”; they just read and/or listened to the speaker voice that only said the symptom statement. This generated confusion and questions about how the symptom statements should be interpreted. These assessment items were evaluated and further developed at a third evaluation with children and parents. In addition to suggestions for minor revisions this interaction also showed that it was important that the assessment items did not begin in the same way, for example, “How is this for you...” because it was then perceived as being tedious to answer the assessment items. The varying of the assessment items also entailed changes to the first response on the Likert scale, from “No problem” to “No/No problem” (Figure 4).

In addition, some animations contained 2 symptoms in 1 symptom statement (2 of 82), which reduced clarity. Therefore, these animations were divided into 1 symptom per symptom statement. The parents and the pediatric nurses also questioned whether some symptom statements (6 of 82) were relevant, such as “Act younger than I am.” They maintained that the children were not aware of their behavior in that way. The pediatric nurses and parents also suggested a new assessment item, “How is it for you to get a needle into the port?”, because this was a common procedure in Swedish hospitals. All inputs led to the symptom statements being revised and finally rephrased into 84 assessment items.

At the final high-fidelity evaluation, the children and parents were able to see the final result and how they had contributed to the development and validation of the language and content in Sisom 2. The children confirmed that they found it easier to understand what was asked and how they should respond when the symptoms were presented as assessment items rather than as symptom statements. Some assessment items in Sisom 2 were more generic and applicable in many situations (eg, headaches) and others were more cancer specific (eg, hair loss), but this approach was not perceived as a problem for the children. If the children had no experience of what was presented by the assessment item, they answered it with the smiley “Don't know.” The participants judged the assessment items as relevant and easy to understand, indicating good face and content validity.

### Evaluation of Aesthetics

At the first low-fidelity evaluation of aesthetics, children liked the fact that they were allowed to participate through a child avatar in Sisom and enjoyed the concept of a boat that could travel between different islands. The adjustment of the visual design to suit an iPad, rather than a desktop computer, led to them seeing a potential for choosing different environments for the avatar to travel in. Examples of these are driving a water scooter, car, motorcycle, airplane, tractor, train, kickboard, or riding a bike or a horse, or being a dinosaur, princess, hairdresser, or various animals, or completely changing the environment into a jungle, zoo, farm, and so on. The children would also have liked the opportunity to choose various attributes for the avatar, such as a shawl, a beanie, or a cap and various hairstyles, which was accommodated. Some parents wondered whether it was important that the children had to choose to be a boy or a girl in the introduction. They pointed out that the avatar was gender neutral and that the children themselves could determine what the avatar would look like, with the help of skin color, headdress, and clothes. The feedback on attributes for the avatar was incorporated in the visual design, whereas the suggestions for different environments and a gender-neutral avatar composition were considered to be beyond the scope of the ambitions for Sisom 2. These could, however, be important improvements for future versions of Sisom and be used as functionalities that can drive children’s intrinsic motivation to use Sisom over longer periods of time.

**Figure 5:**
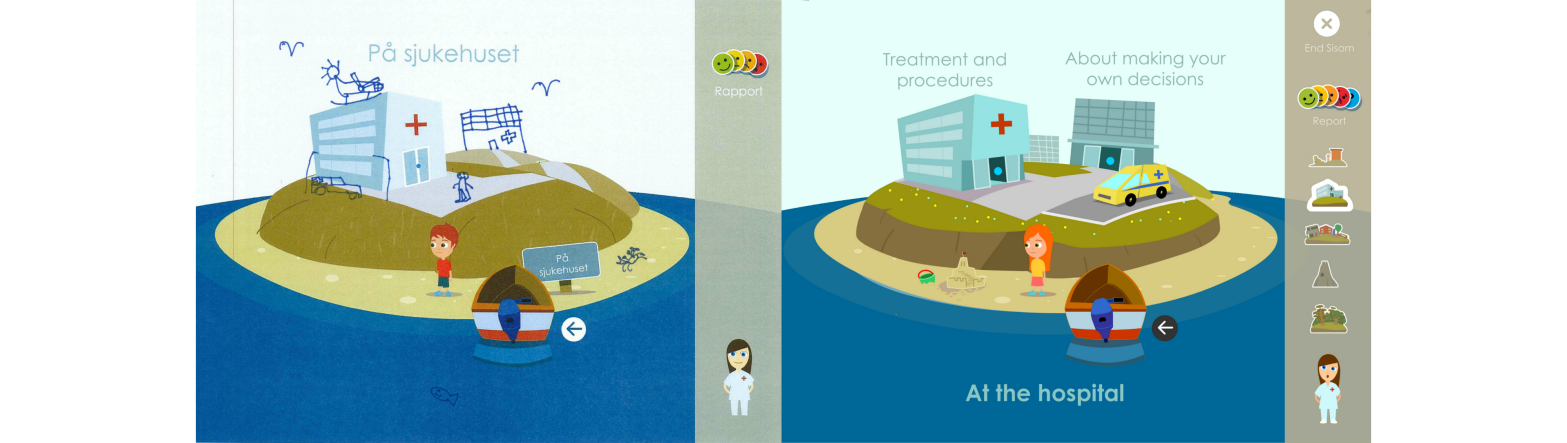
The children suggested that the pictures in Sisom should have more details. Left: an example of what a child drew on a screenshot; Right: the final version in Sisom 2.

The children did not think Sisom was as fun as an ordinary iPad game at the first low-fidelity evaluation. They said, “The colors in Sisom are boring and gloomy.” The children and parents also thought it was important that positive and exciting things appeared. They preferred brighter colors and more details, such as fishes in the sea, birds flying, clouds, crabs on the beaches, a sand castle, an ambulance and helicopter outside the hospital, and so on (Figure 5). The children would also like to hear background noises from the birds and other things that appeared visually. These suggestions were embedded in the next version of Sisom.

**Figure 6:**
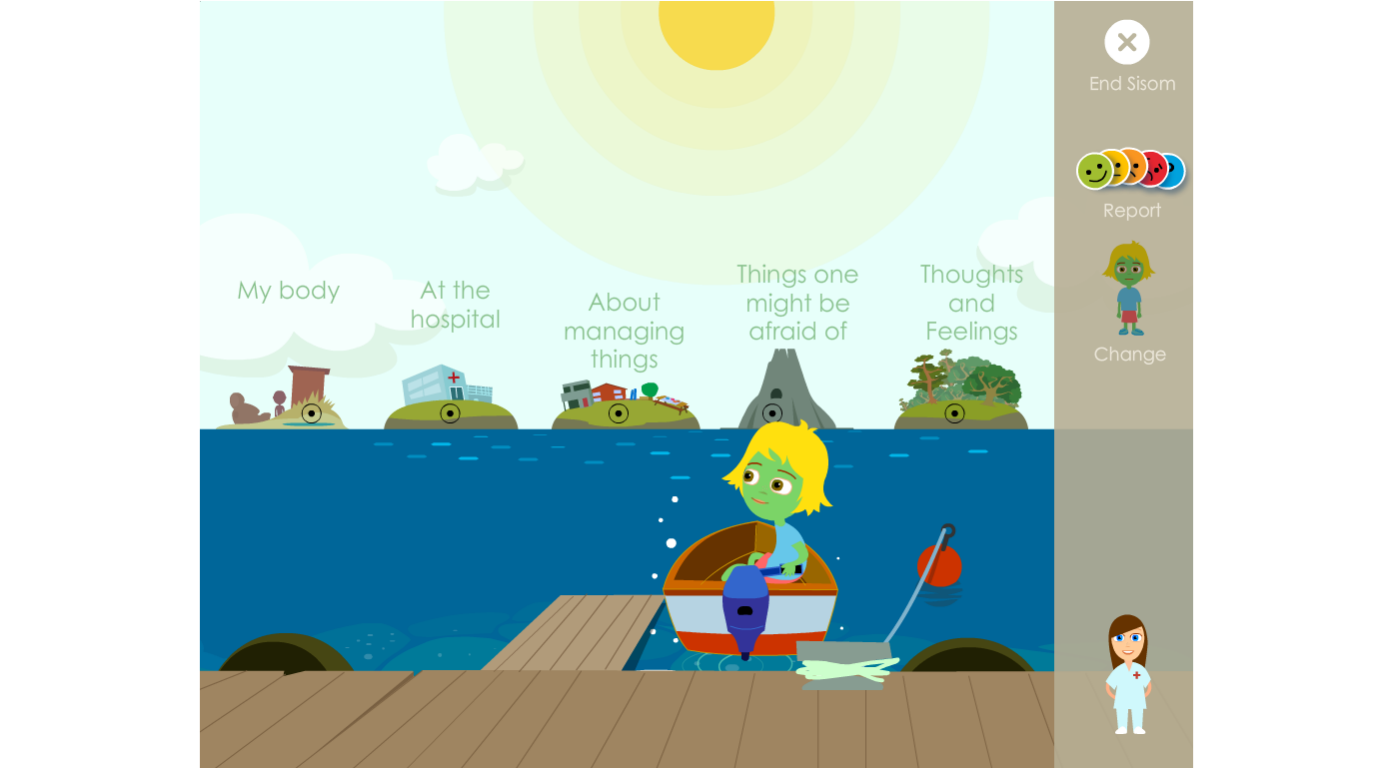
This start view shows the refinements based on the children’s suggestions of brighter colors and more details.

At the second evaluation the children and parents thought that the animations in Sisom 2 had become much better and that the colors were happier and brighter. They liked that their suggestions had been taken into account when changes in Sisom had been made (Figure 6).

**Figure 7:**
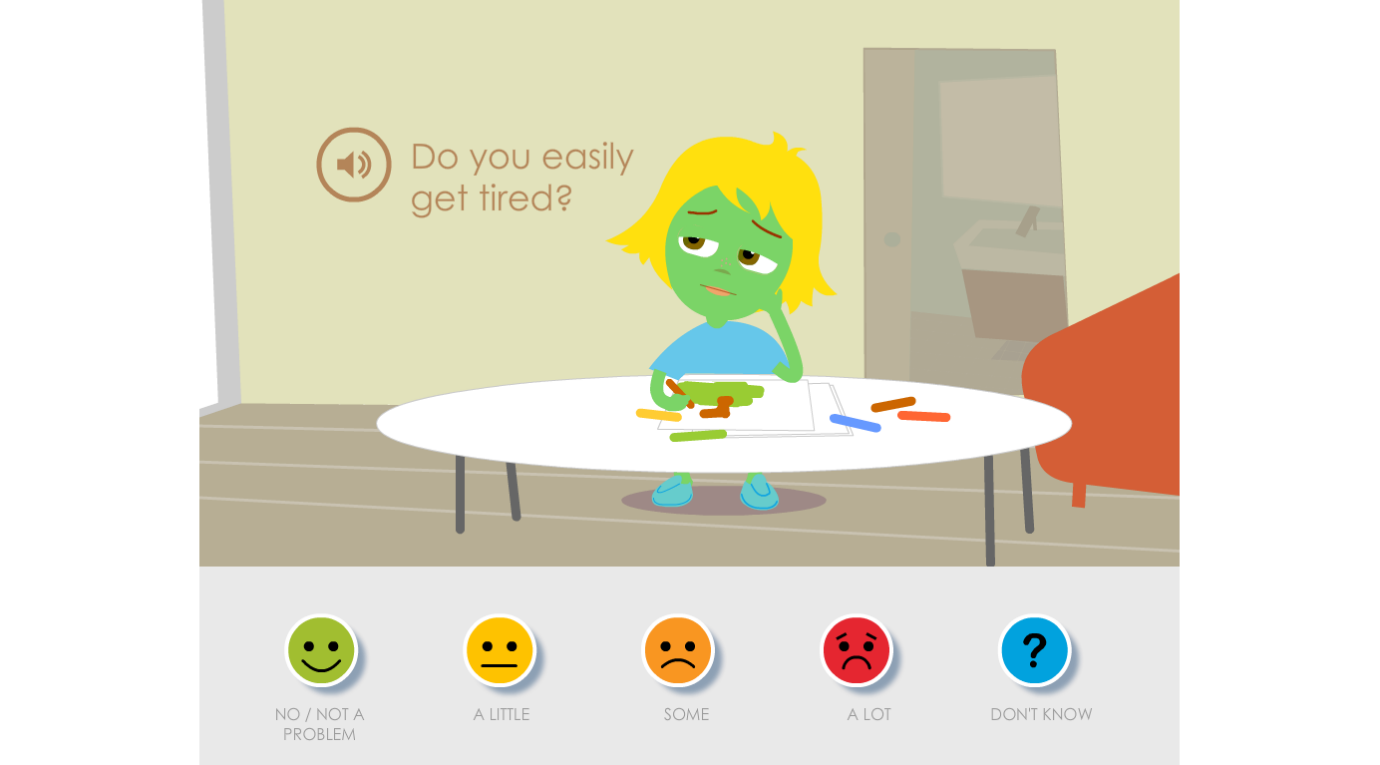
This picture shows the refinements based on the children and parents’ suggestions to change the open mouth of the red smiley to a more sad illustration.

At the first low-fidelity evaluation it emerged that the children, who had not read the text beneath the smileys, had not understood the difference between the smileys in orange and red in the Likert scale. They thought the only difference was that the mouth was open in the red smiley. At the next evaluation the mouth of the red smiley was changed to be sadder and this was better according to both children and parents (Figures 2 and 7).

**Figure 8:**
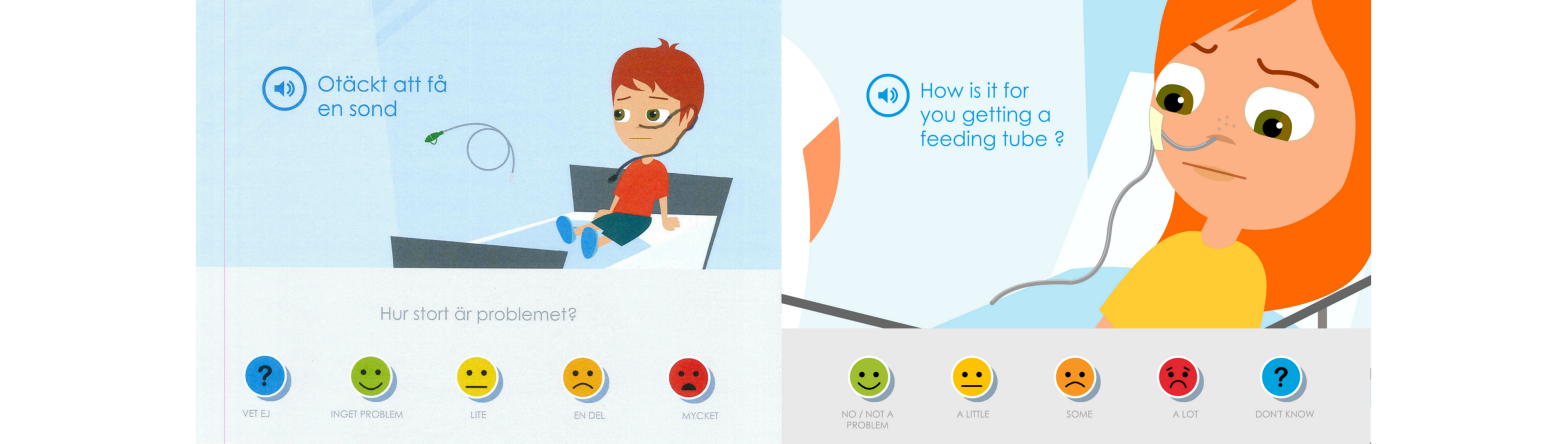
The children wanted the object in the pictures to be integrated with the avatar in the animation and not be “in the air” beside the avatar. Left: an example of what a child drew on a screenshot; Right: the final version in Sisom 2.

On the “At the hospital” island the symptoms contained medical supplies such as a syringe, a tube, and so on. In order to avoid the children being intimidated by this equipment the design and programming had been simplified so that these objects appeared as symbols detached from their proper use in the animations. The children suggested, however, that the objects ought to be integrated into the animations and involve the avatar instead of being placed “in the air” beside the avatar, for example, as in the picture “Disgusting to get a feeding tube” (Figure 8).

**Figure 9:**
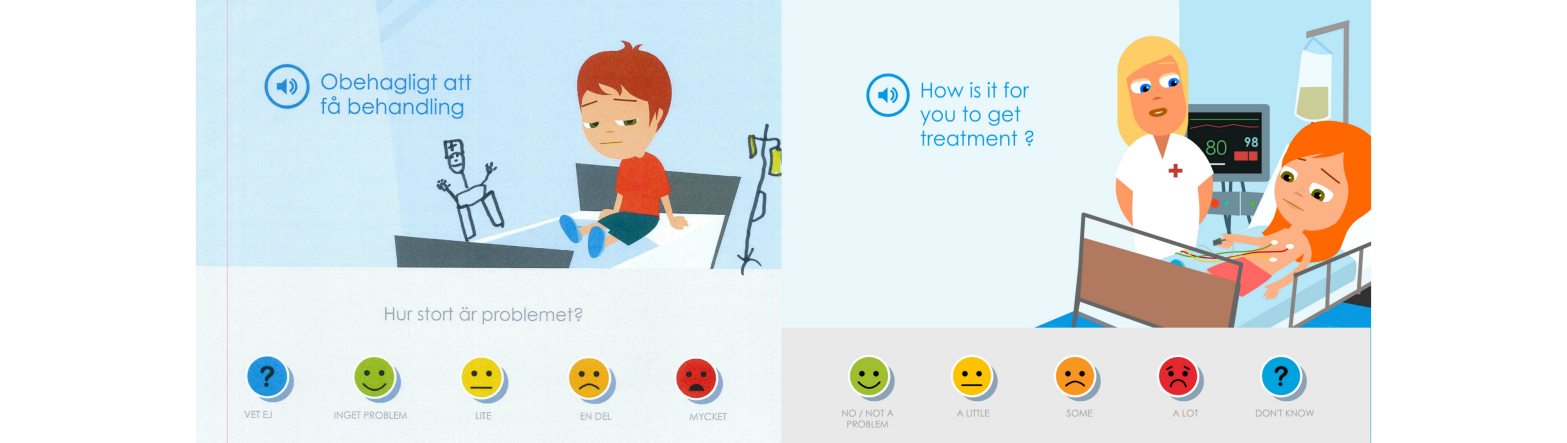
The children suggested that pictures at the hospital should include a nurse or a doctor. Left: an example of what a child drew on a screenshot; Right: the final version in Sisom 2.

The children also thought it would be better if some animations, for example, the picture with the symptom statement “To get shots,” were illustrated by a cartoon nurse holding the shots and with the nurse standing beside the avatar. The children also thought that there should be a nurse or a doctor in the treatment room at the hospital (Figure 9) and a teacher in the classroom at school. For the next evaluations, these changes were made iteratively until the children thought it was better and clearer (Figure 9).

**Figure 10:**
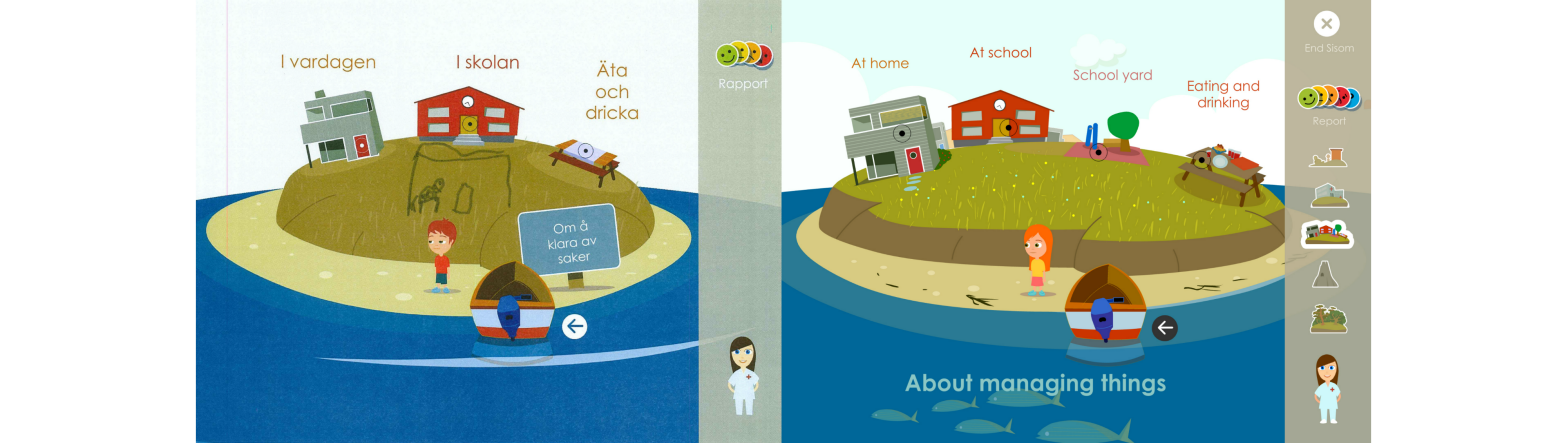
The children suggested that a schoolyard should be included at the school. Left: an example of what a child drew on a screenshot; Right: the final version in Sisom 2.

At each evaluation, children were very good at detecting whether there were missing details in the pictures. For example, in the animation for “Eat and drink,” a child thought there should be a missing pizza slice in the box because there was a pizza slice on the plate. Another example was that children thought that the animation for “Often thirsty” would have been better if the avatar drank from several glasses instead of from the pitcher. The children and parents also suggested new topics that they thought should be included in Sisom 2. One example was to include a schoolyard at the school (Figure 10), because the children had sometimes experienced problems during breaks between lessons.

In the first version of the “My body” island, all parents thought that one of the sandmen looked “grotesque,” especially since the island was concerned with problems that the children had with their bodies. The parents wondered how a child, who had experienced surgery to amputate a part of the body, would feel when answering these assessment items. None of the children reacted to the sandman's appearance, but when they saw the modified version based on the parents’ input, they thought that it was much better and that the new details “were cool.”

**Figure 11:**
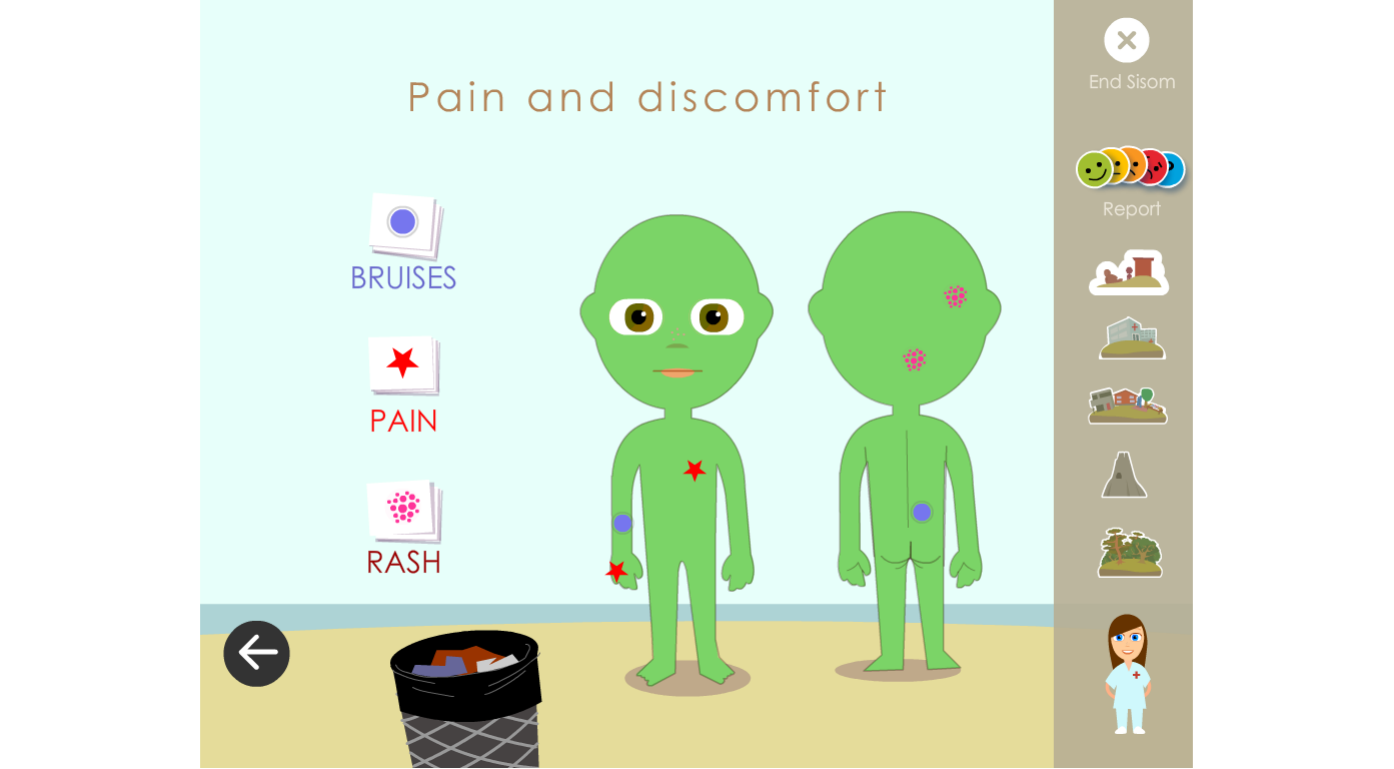
The children appreciated being able to answer by indicating areas of pain, bruises, and rashes on a body map.

When the children answered the assessment items about “Pain and discomfort,” they were considerably more engaged and happy with the way they could report their experiences. These assessment items were answered by indicating areas of pain, bruises, and rashes on a body map using color-specific icons (Figure 11). The responses of the children and parents were similar to previous comments about them preferring variations in how the children could answer assessment items.

There were 84 symptoms in the final version of Sisom 2, which were individually represented by assessment items and animations, and more than 100 large or small visual modifications had been made based on the participants’ views. At the concluding high-fidelity evaluation when the children compared Sisom 2 with other games on their own iPads, they thought the animations in Sisom 2 should remain being on a simpler level and not so realistic. If Sisom 2 had been more lifelike, then many images, such as removing stitches, would be too frightening. The children also thought that it was easier to answer the assessment items in Sisom 2 compared with answering the same assessment items in a paper questionnaire or orally, because in Sisom 2 they received guidance from the animations on how to interpret the assessment items.

### Evaluation of Usability

At the first low-fidelity evaluation of usability, the children and parents requested an integration of more support and instructions in order to reduce uncertainties in understanding statements and animations correctly and to increase the usability. As verbal and visual support and instructions were inserted, both children and parents thought clarity was increased. However, the need for instructions decreased as the children became familiar with the system. One child said, “Can’t stand to listening to the instructions all the time.” They thought it was a major improvement, at the next evaluation, when the verbal instructions appeared only once after they had answered all assessment items on an island. The voice representing the cartoon nurse was of great importance for the children and parents’ experiences of Sisom 2. At the first evaluations they wished for a happier and more stimulating voice, and after the revision they thought that the voice sounded less sad and more “Normal, like someone you know.”

The children with experiences of cancer treatment, the parents, and pediatric nurses thought that the children would not have had the strength or concentration to manage to answer all assessment items in Sisom 2 in some phases of the disease and treatment. They thought it was thus important that answers to the assessment items in Sisom 2 could be saved and that they could pause and continue with the remaining assessment items later on without having to start from the beginning.

The comments from the parents and pediatric nurses together with the researchers’ observations about how the children navigated in Sisom contributed to technical difficulties and bugs being identified and resolved. All participants then acknowledged the usability and qualities of using the final version of Sisom 2 in the clinical work at pediatric clinics at the concluding high-fidelity evaluation. They thought that the system was usable and would consider using it if offered. Both pediatric nurses and parents considered the final versions of the assessment items applicable for children with many different conditions and not cancer diagnosis specific.

## Discussion

New mobile devices provide powerful tools for children in health care, with the potential for paying attention to their needs. This can change the communication pattern between children and health care professionals, as well as strengthen children’s empowerment [11,29]. We, therefore, redesigned and validated Sisom in order to meet the new opportunities for adaptation to mobile devices, with the purpose of it being applied as a user-friendly assessment and communication tool in pediatric care. The user-experience approach to redesign and validate Sisom in this study was guided by the evaluation of content, aesthetics, and usability.

As found in earlier research [30], the children in our study described that it was easier and more fun to answer assessment items in an animated application than in a paper questionnaire or orally. Earlier research has shown that children’s experiences of being asked questions in hospital settings are generally not positive, because they are only asked a few specific questions, primarily about symptoms, and then the health care professionals discuss the issue with their parents [7]. Children’s preferences for participation vary and it is important that their views are sought on how they want to communicate and be involved in their own health care [7,31]. Our intention is that Sisom 2 will be a communication tool that is adapted to the new technology in order to capture children’s own needs and preferences in health care.

Children from a very young age are now generally comfortable with mobile devices, such as an iPad, mobile phone, and tablet devices [32,33]. However, how the language is used in the instructions and tasks during the play is important because this may affect children’s opportunities to understand the instructions and tasks that are given to them [34,35]. A mixture of audio and textual instructions in the mobile devices seems to be most suitable to support the children’s different levels of ability and preference [36,37]. In this study, major improvements were made regarding both the verbal instruction of how to navigate and how to ask assessment items in Sisom 2 to the child. In the original version of Sisom the assessment items were pronounced as symptom statements and then the children answered these symptom statements based on the assessment item “How much of a problem?” The children had difficulties understanding this way of answering because of confusion as to how the symptom statement and the assessment items were connected. This led to Sisom 2 containing only 1 assessment item for each symptom and the assessment item is the same in both written text and in the audio from the cartoon nurse. Previous research has shown that young schoolchildren prefer statements rather than assessment items [38]; however, the biggest problem in our study was that the symptom contained both a symptom statement and an assessment item and that the written text and sounds not were the same. The assessment items were also changed in order to be more neutral to avoid assumptions that the symptom already was a problem. The reason for the suggested improvements of the assessment items was to not take for granted that the symptom was a problem for the child. It is more appropriate to ask the child how he or she feels about the symptom. Having a more salutogenic perspective in Sisom 2 could provide health care professionals with the possibility of paying attention to the children’s own defined health experiences, instead of focusing on predefined problems related to the disease [39]. The modification of problem-oriented statements to more salutogenic or neutral assessment items in Sisom 2 is in line with the recommendation that the language in health care should shift from disease to health in order to improve the conditions for empowering patients [40-42].

Children want apps on mobile devices to be appealing and fun to use [22,43]. In this study the children suggested substantial improvements in the colors, sounds, and animations in order to have more fun when answering the assessment items. The children’s preferences on the aesthetics in the digital environment seemed to be equivalent to their preferences in the hospital environment. In earlier research children described that brighter colors in the hospital environment contribute to positive emotions and darker colors contribute to negative and stressed emotions [44-46]. Both visual and auditory effects were important to maintain the children's interest and motivate them to go through the whole Sisom 2. However, the animations should be of a simpler nature because otherwise some assessment items concerning difficult disease-related issues, for example, if they were afraid of dying, could be perceived as scary or as inappropriate. Other preferences from the children was that they thought it would be fun if they could travel around in Sisom in different ways and if the environment could change to other sceneries other than traveling by boat between islands. Such alternatives were seen as ways of increasing enthusiasm and intrinsic motivation to respond to Sisom 2 several times and during a period of illness. This is an important aspect for further development of Sisom 2, for uses in settings where motivation is a factor that can limit usefulness and feasible implementation; however, this was beyond the scope of this study and for the redesign of Sisom.

The final version of Sisom 2 made it technically feasible for the child to pause “the game” if they did not manage to complete all assessment items at the same time. The data that they had already filled in were stored. This is an important technical improvement because one common problem for children with cancer, as well for other diagnoses, is increased fatigue during the treatment [47-50]. Many children in health care have complex communication needs, which require technical support in order to convey how they feel. We anticipate that the redesign of Sisom 2 allows for more generic and non–diagnosis-specific use. Studies of Sisom 2 thus need to evaluate how children (aged 6-12 years) with different life-threatening, lifelong, or prolonged diseases find Sisom 2 usable as an interactive assessment and communication tool. There is also a need to investigate how Sisom 2 as a new digital service in pediatric care can transform the way health care professionals deliver health care as well as if Sisom 2 can strengthen children’s participation in their own care.

### Methodological Considerations

An important challenge in research with children is to find appropriate ways for engaging and creating opportunities for children to have genuine influence on the research process [51]. Children in our study have added valuable views and quality of ideas regarding content, aesthetics, and usability that have genuinely influenced the design of Sisom 2. Involving children throughout the low- and high-fidelity evaluation was considered as essential for the outcomes.

A key factor in any evaluation is the quality of the target group representation during investigations. In this study we have involved fairly few children, parents, and health care professionals in repeated encounters. On the other hand their involvement was extensive and together they represented healthy children with some experiences of medical treatment, children with experiences of cancer treatment, their parents, and pediatric nurses and thus contributed with valuable different experiences and perspectives. Research has shown that 80% of all usability problems are detected with as few as 4-5 participants [52], and we are therefore inclined to believe that the number of participants in our study was sufficient to address the usability issues we wanted to evaluate. The healthy children with some experiences of medical treatment were used as proxies for children with experiences of cancer treatment and were recruited from academic researchers’ families, which may be a bias.

Ruland et al [16] have shown that healthy children can only partially conceptualize what it is like suffering from a serious illness, and thus the degree to which they can serve as proxies in participatory design and evaluations is limited. However, Ruland et al [16] thought that personal experiences could be an important factor for valuable design contributions. All the healthy children in this study had some experience of medical treatment (eg, surgery, vaccination, treatment of eczema) and their descriptions of the general aspects of content, aesthetics, and usability in Sisom 2 were compatible with the description from the children with experiences of cancer treatment. Another important design issue is that all the children were white and ethnically Swedish. Therefore, an important next step is to refine and adjust Sisom 2 to children from different ethnic backgrounds.

One aspect that was missing, however, was that the parents’ perspectives were almost entirely presented by the mothers, and the fathers were consulted by the mothers either only occasionally or rarely and briefly participated in the discussions. We do not know how important more contribution of the fathers or children during ongoing treatment at the hospital setting could have been. Children with cancer who were involved in ongoing treatment were not included in the study because of ethical considerations and because they were often included in several different studies.

### Conclusions

In conclusion, this study illuminates the process of using a user-experience design with children to redesign and validate the interactive assessment and communication tool Sisom, and it describes how the evaluation of content, aesthetics, and usability resulted in a revised version, Sisom 2. Involving children throughout this research process was essential for including valuable views relevant for the user group and to ascertain quality of ideas. These contributions genuinely influenced the design of Sisom 2. The modification of problem-oriented statements to more salutogenic assessment items was the most important revision of the content in Sisom 2. Parents and pediatric nurses considered the revised assessment items to be less diagnosis specific. The evaluation of the aesthetics resulted in brighter colors and more positive and exciting details in the animations. The evaluation of usability improved the verbal instructions for how to navigate, while also enabling the answers to assessment items in Sisom 2 to be saved so that the children could pause and continue to answer the remaining assessment items at a later stage. All participants confirmed the usability and qualities of using the final version of Sisom 2. Future research should be directed toward studying the implementation of Sisom 2 in various clinical practice contexts and its effects on patient care and outcomes.

## References

[1] Steliarova-Foucher E, Ullrich A (2015). WHO.

[2] Darcy L, Knutsson S, Huus K, Enskär K (2014). The everyday life of the young child shortly after receiving a cancer diagnosis, from both children's and parent's perspectives. Cancer Nurs.

[3] Coyne I, Amory A, Kiernan G, Gibson F (2014). Children's participation in shared decision-making: children, adolescents, parents and healthcare professionals' perspectives and experiences. Eur J Oncol Nurs.

[4] Lipstein EA, Brinkman WB, Sage J, Lannon CM, Morgan DeWitt E (2013). Understanding treatment decision making in juvenile idiopathic arthritis: a qualitative assessment. Pediatr Rheumatol Online J.

[5] Coyne I, Kirwan L (2012). Ascertaining children's wishes and feelings about hospital life. J Child Health Care.

[6] Vaknin O, Zisk-Rony RY (2011). Including children in medical decisions and treatments: perceptions and practices of healthcare providers. Child Care Health Dev.

[7] Coyne I, Gallagher P (2011). Participation in communication and decision-making: children and young people's experiences in a hospital setting. J Clin Nurs.

[8] Virkki M, Heino Tolonen T, Koskimaa T, Paavilainen E (2015). Children as decision-makers in health care - an integrative review. Clinical Nursing Studies.

[9] Noreña Peña AL, Rojas JG (2014). Ethical aspects of children's perceptions of information-giving in care. Nurs Ethics.

[10] Wiering BM, Noordman J, Tates K, Zwaanswijk M, Elwyn G, De Bont ES, Beishuizen A, Hoogerbrugge PM, Van Dulmen S (2016). Sharing decisions during diagnostic consultations; an observational study in pediatric oncology. Patient Educ Couns.

[11] McNaughton D, Light J (2013). The iPad and mobile technology revolution: benefits and challenges for individuals who require augmentative and alternative communication. Augment Altern Commun.

[12] (1990). United Nation.

[13] Söderbäck M, Coyne I, Harder M (2011). The importance of including both a child perspective and the child's perspective within health care settings to provide truly child-centred care. J Child Health Care.

[14] Coyne I, Harder M (2011). Children's participation in decision-making: balancing protection with shared decision-making using a situational perspective. J Child Health Care.

[15] Ruland CM, Slaughter L, Starren J, Vatne TM, Moe EY (2007). Children's contributions to designing a communication tool for children with cancer. Stud Health Technol Inform.

[16] Ruland CM, Starren J, Vatne TM (2008). Participatory design with children in the development of a support system for patient-centered care in pediatric oncology. J Biomed Inform.

[17] Vatne TM, Finset A, Ørnes K, Holmstrøm H, Småstuen MC, Ruland CM (2013). Effects of an interactive symptom communication tool for children with heart disease on patient-provider communication in outpatient care: Preliminary results. Journal of Communication in Healthcare.

[18] Vatne TM, Slaugther L, Ruland CM (2010). How children with cancer communicate and think about symptoms. J Pediatr Oncol Nurs.

[19] Baggott C, Baird J, Hinds P, Ruland CM, Miaskowski C (2015). Evaluation of Sisom: A computer-based animated tool to elicit symptoms and psychosocial concerns from children with cancer. Eur J Oncol Nurs.

[20] Tsimicalis A, Stone PW, Bakken S, Yoon S, Sands S, Porter R, Ruland C (2014). Usability testing of a computerized communication tool in a diverse urban pediatric population. Cancer Nurs.

[21] Druin A (2010). Children as codesigners of new technologies: valuing the imagination to transform what is possible. New Dir Youth Dev.

[22] Stinson JN, Jibb LA, Nguyen C, Nathan PC, Maloney AM, Dupuis LL, Gerstle JT, Alman B, Hopyan S, Strahlendorf C, Portwine C, Johnston DL, Orr M (2013). Development and testing of a multidimensional iPhone pain assessment application for adolescents with cancer. J Med Internet Res.

[23] Ruland CM, Hamilton GA, Schjødt-Osmo B (2009). The complexity of symptoms and problems experienced in children with cancer: a review of the literature. J Pain Symptom Manage.

[24] (2016). World Health Organization.

[25] Chen H, Boore JRP (2010). Translation and back-translation in qualitative nursing research: methodological review. J Clin Nurs.

[26] Maneesriwongul W, Dixon JK (2004). Instrument translation process: a methods review. J Adv Nurs.

[27] Van Someren MW, Barnard YF, Sandberg JAC (1994. ISBN 0-12-714270-3). The think aloud method: a practical guide to modelling cognitive processes.

[28] Krippendorff K (2013). Content analysis. An introduction to its methodology.

[29] Raaff C, Glazebrook C, Wharrad H (2014). A systematic review of interactive multimedia interventions to promote children's communication with health professionals: implications for communicating with overweight children. BMC Med Inform Decis Mak.

[30] De Leo G, Gonzales CH, Battagiri P, Leroy G (2011). A smart-phone application and a companion website for the improvement of the communication skills of children with autism: clinical rationale, technical development and preliminary results. J Med Syst.

[31] Coyne I, Amory A, Gibson F, Kiernan G (2016). Information-sharing between healthcare professionals, parents and children with cancer: more than a matter of information exchange. Eur J Cancer Care (Engl).

[32] Kabali HK, Irigoyen MM, Nunez-Davis R, Budacki JG, Mohanty SH, Leister KP, Bonner RL (2015). Exposure and Use of Mobile Media Devices by Young Children. Pediatrics.

[33] Stålberg A, Sandberg A, Söderbäck M, Larsson T (2016). The child's perspective as a guiding principle: Young children as co-designers of an interactive application to facilitate participation in healthcare situations. J Biomed Inform.

[34] Lieberman DA, Ritterfield MC, Vorderer P (2009). Designing serious games for learning and health in informal and formal settings. Serious games: Mechanisms and effects.

[35] Chin J, Mengping T (2014). A Multi-modal digital game-based learning environment for hospitalized children with chronic illnesses. Educational Technology & Society.

[36] McKnight L, Fitton D (2010). Touch-screen technology for children: giving the right instructions and getting the right responses.

[37] Hiniker A, Sobel K, Hong SR, Suh H, Irish I, Kim D, Kientz JA (2015). Touchscreen prompts for pre-schoolers: Designing developmentally appropriate techniques for teaching young children to perform gestures.

[38] Lassetter JH, Ray G, Driessnack M, Williams M (2015). Consulting with children in the development of self-efficacy and recall tools related to nutrition and physical activity. J Spec Pediatr Nurs.

[39] Antonovsky A (1996). The salutogenic model as a theory to guide health promotion. Health Promot Int.

[40] Hollnagel H, Malterud K (2000). From risk factors to health resources in medical practice. Med Health Care Philos.

[41] Anderson RM, Funnell MM (2010). Patient empowerment: myths and misconceptions. Patient Educ Couns.

[42] Groen WG, Kuijpers W, Oldenburg HS, Wouters MWJM, Aaronson NK, Van Harten WH (2015). Empowerment of Cancer Survivors Through Information Technology: An Integrative Review. J Med Internet Res.

[43] Stinson J, McGrath P, Hodnett E, Feldman B, Duffy C, Huber A, Tucker L, Hetherington R, Tse S, Spiegel L, Campillo S, Gill N, White M (2010). Usability testing of an online self-management program for adolescents with juvenile idiopathic arthritis. J Med Internet Res.

[44] Coad J, Coad N (2008). Children and young people's preference of thematic design and colour for their hospital environment. J Child Health Care.

[45] Sjöberg C, Amhliden H, Nygren JM, Arvidsson S, Svedberg P (2015). The perspective of children on factors influencing their participation in perioperative care. J Clin Nurs.

[46] Burkitt E, Sheppard L (2013). Children's colour use to portray themselves and others with happy, sad and mixed emotion. Educational Psychology.

[47] Perdikaris P, Merkouris A, Patiraki E, Tsoumakas K, Vasilatou-Kosmidis E, Matziou V (2009). Evaluating cancer related fatigue during treatment according to children's, adolescents' and parents' perspectives in a sample of Greek young patients. Eur J Oncol Nurs.

[48] Wu M, Hsu L, Zhang B, Shen N, Lu H, Li S (2010). The experiences of cancer-related fatigue among Chinese children with leukaemia: a phenomenological study. Int J Nurs Stud.

[49] Butbul Aviel Y, Stremler R, Benseler SM, Cameron B, Laxer RM, Ota S, Schneider R, Spiegel L, Stinson JN, Tse SML, Feldman BM (2011). Sleep and fatigue and the relationship to pain, disease activity and quality of life in juvenile idiopathic arthritis and juvenile dermatomyositis. Rheumatology (Oxford).

[50] Maher C, Crettenden A, Evans K, Thiessen M, Toohey M, Watson A, Dollman J (2015). Fatigue is a major issue for children and adolescents with physical disabilities. Dev Med Child Neurol.

[51] Wärnestål P, Svedberg P, Nygren J (2014). Co-constructing Child Personas for Health-Promoting Services with Vulnerable Children.

[52] Virzi RA (1992). Refining the test phase of usability evaluation: how many subjects is enough?. Hum Factors.

